# Assessing quality of care for the dying from the bereaved relatives’ perspective: Using pre-testing survey methods across seven countries to develop an international outcome measure

**DOI:** 10.1177/0269216318818299

**Published:** 2019-01-10

**Authors:** Catriona Rachel Mayland, Christina Gerlach, Katrin Sigurdardottir, Marit Irene Tuen Hansen, Wojciech Leppert, Andrzej Stachowiak, Maria Krajewska, Eduardo Garcia-Yanneo, Vilma Adriana Tripodoro, Gabriel Goldraij, Martin Weber, Lair Zambon, Juliana Nalin Passarini, Ivete Bredda Saad, John Ellershaw, Dagny Faksvåg Haugen

**Affiliations:** 1 Palliative Care Institute, Cancer Research Centre, University of Liverpool, Liverpool, UK; 2 Department of Oncology & Metabolism, University of Sheffield, Sheffield, UK; 3 Interdisciplinary Palliative Care Unit, Department of Medicine, University Medical Center of Johannes Gutenberg University Mainz, Mainz, Germany; 4 Regional Centre of Excellence for Palliative Care, Western Norway, Haukeland University Hospital, Bergen, Norway; 5 Haraldsplass Deaconess Hospital, Bergen, Norway; 6 Department of Clinical Medicine (K1), University of Bergen, Bergen, Norway; 7 Department of Palliative Medicine, Poznan University of Medical Sciences, Poznan, Poland; 8 Department of Quality of Life Research, Medical University of Gdansk, Gdansk, Poland; 9 Sue Ryder House, Pallmed, Bydgoszcz, Poland; 10 Mutualista Hospital Evangélico, Montevideo, Uruguay; 11 Pallium Latinoamérica, Buenos Aires, Argentina; 12 Instituto de Investigaciones Medicas Alfredo Lanari, University of Buenos Aires, Buenos Aires, Argentina; 13 Hospital Privado Universitario de Córdoba, Córdoba, Argentina; 14 Department of Internal Medicine, Campinas State University, Campinas, Brazil; 15 Sumaré State Hospital, Campinas, Brazil; 16 Academic Palliative and End-of-Life Care Department, Royal Liverpool University Hospital, Liverpool, UK

**Keywords:** Terminal care, quality of healthcare, proxy, survey and questionnaire, cognitive interviewing, quality of care for the dying

## Abstract

**Background:**

The provision of care for dying cancer patients varies on a global basis. In order to improve care, we need to be able to evaluate the current level of care. One method of assessment is to use the views from the bereaved relatives.

**Aim:**

The aim of this study is to translate and pre-test the ‘Care Of the Dying Evaluation’ (CODE^TM^) questionnaire across seven participating countries prior to conducting an evaluation of current quality of care.

**Design:**

The three stages were as follows: (1) translation of CODE in keeping with standardised international principles; (2) pre-testing using patient and public involvement and cognitive interviews with bereaved relatives; and (3) utilising a modified nominal group technique to establish a common, core international version of CODE.

**Setting/participants:**

Hospital settings: for each country, at least five patient and public involvement representatives, selected by purposive sampling, fed back on CODE^TM^ questionnaire; and at least five bereaved relatives to cancer patients undertook cognitive interviews. Feedback was collated and categorised into themes relating to clarity, recall, sensitivity and response options. Structured consensus meeting held to determine content of international CODE (i-CODE) questionnaire.

**Results:**

In total, 48 patient and public involvement representatives and 35 bereaved relatives contributed to the pre-testing stages. No specific question item was recommended for exclusion from CODE^TM^. Revisions to the demographic section were needed to be culturally appropriate.

**Conclusion:**

Patient and public involvement and bereaved relatives’ perceptions helped enhance the face and content validity of i-CODE. A common, core international questionnaire is now developed with key questions relating to quality of care for the dying.

**What is already known about the topic?**

- The Quality of Death Index showed variability in the international provision of care for the dying.
- In order to improve care, we need to have validated outcome measures to assess the current quality of care.
- One method of evaluation is to use the views from the bereaved relatives to assess their own perceptions and as proxy measures for the patient.

**What this paper adds?**

- We have developed a common, core international ‘Care Of the Dying Evaluation’ (i-CODE) questionnaire, assessing both patient care and family-carer support.
- Engagement of patient and public representatives and bereaved relatives has informed the development process adding to the face and content validity of i-CODE.

**Implications for practice, theory or policy**

- i-CODE will enable a transnational comparison of care for the dying to be conducted.
- Results of i-CODE can be used directly for quality improvement purposes.
- i-CODE may be further developed into an international standard and benchmarking tool.

## Background

Providing high quality of care for the dying is fundamentally important and globally remains a key political and economic issue. The provision of care, however, remains diverse. The Quality of Death Index 2015^1^ measures the quality of palliative care across 80 countries. It uses ‘20 quantitative and qualitative indicators across five categories: the palliative and healthcare environment, human resources, the affordability of care, the quality of care, and the level of community engagement’.^1^ Many European countries such as the United Kingdom and Norway fall within the top 30 of this ranking, while other countries such as those within South America have lower positions. A recent report on the current state of palliative and end-of-life care in South America demonstrated that specialist palliative care (SPC) is still not acknowledged as a speciality in 80% of Latin American countries, and hence it is not included within public health services.^2^ A further issue is that only half of patients in the terminal stages of disease receive palliative care. Even within the United Kingdom, the country highest placed on the Quality of Death Index 2015 ranking, there are significant variations in the care for dying patients within English hospitals.^3^ Within Norway (overall ranking of 13), there is a lack of robust measures to evaluate care for dying patients, meaning audit, service evaluation and cross-site comparison are hampered. Therefore, this demonstrates the need and importance for good process and outcome indicators to be in place within healthcare settings.

One internationally recognised method for evaluating care for dying patients is to assess quality of care from the bereaved relatives’ perspective using post-bereavement surveys. These types of evaluations (both postal surveys and telephone interviews) have been a key component in end-of-life care evaluations in several countries,^4–6^ including North America and parts of Europe and were recommended in the UK End of Life Care Strategy.^7^ A previous review identified issues with instruments using ‘satisfaction’ as an outcome measure.^8^ A further systematic review identified ‘Care Of the Dying Evaluation’ (CODE^TM^) as a potential instrument, with some strong psychometric properties, which would benefit from further development and validation.^9^

CODE^TM^ is a 42-item, self-completion, post-bereavement questionnaire, developed and validated within the United Kingdom, focused on both quality of patient care and the level of family-carer support provided in the last days of life and immediate post-bereavement period.^10^ (See supplementary material) CODE^TM^ is a shortened, more user-friendly version of the original instrument, ‘Evaluating Care and Health Outcomes – for the Dying’ (ECHO-D), which was used with over 700 bereaved relatives in hospice and hospital settings. ECHO-D was shown to be valid, reliable and sensitive in detecting inequalities in care and areas of unmet need.^11–13^ CODE^TM^ and ECHO-D are unique as their conceptual basis is formed from the key components recognised as best practice for ‘care of the dying’ (last days of life).^8^ In addition, they can both be used for cancer and non-cancer deaths. Questions include symptom control; communication; nursing and medical care; provision of fluids; place of death; and emotional and spiritual support. CODE^TM^ was a user-representative outcome measure within the Royal College of Physician–led ‘National Care of the Dying Audit – Hospitals’ within the United Kingdom^14^ and formed part of a quality assurance and benchmarking process to evaluate care for the dying across hospices, hospitals and community settings within a specific region of England.^15,16^ In addition, there have been eight requests for CODE^TM^ to be used internationally and over 40 requests for use within the UK healthcare setting.

This article presents the initial work performed within the project, ‘International Care Of the Dying Evaluation (CODE) – quality of care for cancer patients as perceived by bereaved relatives’ (2017–2020),^17^ funded by the Network of the European Union (EU) and the Community of Latin American and Caribbean States (CELAC) on Joint Innovation and Research Activities (ERANet-LAC). The overall aim of this project is to advance the international evidence-base in care for the dying. This involves undertaking a post-bereavement observational study using the CODE^TM^ questionnaire for cancer patients dying in hospital settings across seven European and Latin American countries, England, Norway, Poland, Germany, Argentina, Brazil and Uruguay.

## Aims and objectives

This study aimed to develop and pre-test the existing CODE questionnaire across the seven countries participating in the ERANet-LAC CODE^TM^ project, in keeping with the principles of the European Organisation for Research and Treatment of Cancer (EORTC) guidelines for questionnaire development.^18^

**Figure 1.**
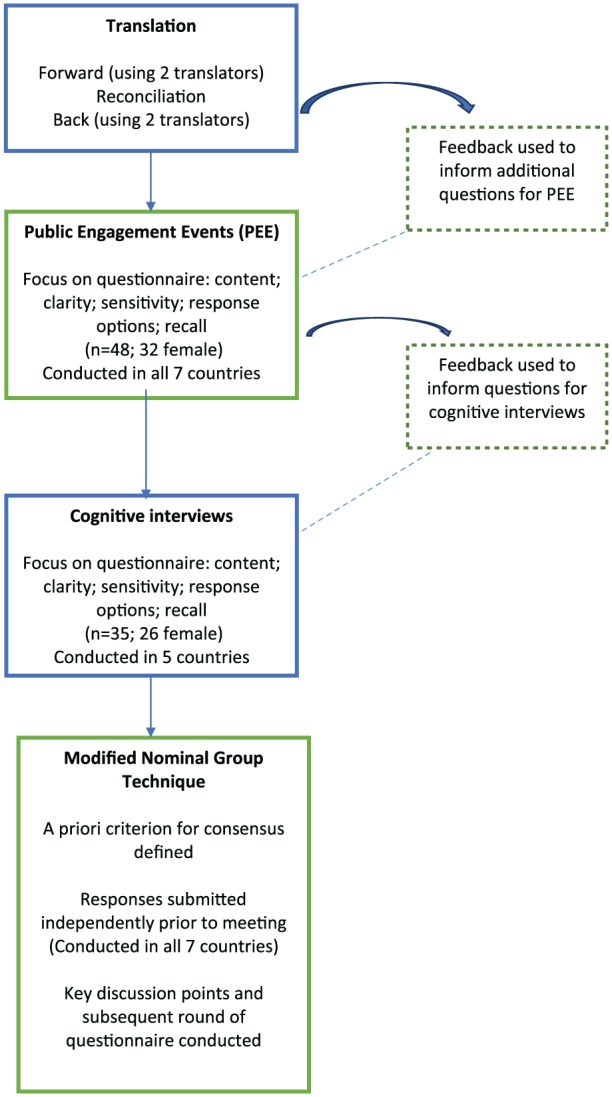
Methods used to develop the international ‘Care Of the Dying Evaluation’ (i-CODE) questionnaire.

The aim was divided into the following objectives:

- Translate CODE^TM^ into the languages used within each of the six non-English countries according to the principles of the EORTC quality-of-life group translation procedure^19^;
- Undertake specific pre-testing of CODE^TM^ using patient and public involvement (PPI) and cognitive interviews with bereaved relatives;
- Utilise a modified nominal group technique^20^ to collate all feedback from the pre-testing and establish a common, core international version of CODE (i-CODE; Figure 1).

## Methods

The study, as a whole, is an observational, cross-sectional, post-bereavement survey using established pre-testing survey methods to develop the questionnaire and then quality improvement methodologies to translate the results into clinical changes. Each part of the research is divided into specific ‘Work Packages’ (WP), namely,

- WP1: Questionnaire development and pre-testing;
- WP2: Conducting post-bereavement survey;
- WP3: Quality improvement work based on questionnaire results.

This article describes the work performed in WP1. The appropriate ethical and institutional approvals were obtained within each country.

### Translation of the CODE^TM^ questionnaire

For each of the languages, the following principles were used: forward translation to native language; reconciliation; and back translation.^19^ This led to preliminary translations which were subsequently proof-read. The German and Polish translation processes had been conducted prior to the commencement of this project.

### Public engagement events

Each country identified at least five participants by purposive sampling, that is, hospital volunteers or representatives from PPI forums, and facilitated a public engagement event. The sample was purposive as we wanted to gain views from those who had experience of care for dying patients; ensure that there was male representation; and in addition, some specific sub-groups were targeted within certain countries, for example, Turkish volunteers in Germany. Ahead of the meeting, potential participants were given a copy of the CODE^TM^ questionnaire; a copy of the letter of approach that would be used within the subsequent international survey; and an outline of the proposed methods for the international survey. With verbal consent, non-identifiable demographic details (gender and role) about the group were collected. In order to ensure consistency, an overarching template was used to direct the format of these events within each country. The project lead (or a nominated delegate) for each country led the event and was supported by other facilitators who were healthcare professionals (with experience in palliative care).

Within the meeting, using a structured question format facilitated by a healthcare professional, participants were asked to feedback about the following:

- The CODE^TM^ questionnaire in terms of format, layout and clarity;
- Individual questions in terms of clarity, sensitivity, ability to recall information to provide a response and use of the response options;
- Any additional question items that should be contained.

In addition, for specific countries (United Kingdom, Germany and Argentina), participants were asked about the letter of approach and to comment on the clarity, appropriateness and sensitivity of the wording. Finally, their views about the proposed methods and conduct of the international survey for their country were sought.

Where possible, the event was audio-recorded and a verbatim transcription produced (in the country’s native language). For all events, a thematic approach was used to analyse the findings with special attention to additional and divergent issues.^21^ Conclusions were translated into English.

### Pre-testing cognitive ‘think aloud’ interviews with bereaved relatives

Questionnaire pre-testing helps assess questionnaire comprehension, relevance and flow. One method is cognitive ‘think aloud’ interviewing.^22^ This involves training respondents to articulate their thoughts as they read a question; recall from their memories the information required; and turn the information they have into an answer.^23^ This provides an understanding of the cognitive processes used to formulate answers and checks how questions have been interpreted.^24^

#### Participants

Due to ethical sensitivities, a purposive sample of potential participants was included according to the following criteria: next-of-kin to an adult patient (18 years or above) who died from cancer in a hospital setting; and over 18 years of age and able to give informed consent. Exclusion criteria were as follows: patient had a sudden, unexpected death; next-of-kin experienced a bereavement within the last 6–8 weeks; or the research team perceived the individual would be unduly distressed by participation. For each new language, a minimum of five bereaved relatives were included. Extensive pre-testing cognitive interviews had already been conducted in English prior to this work.^10,11^

#### Method of approach

An opt-in method was adopted, whereby each potential participant was sent or given a letter of invitation and information pack asking if they would be willing to participate in the study. Within the information pack, a participant information sheet, consent form and response form were included. A member of the research team contacted those who returned the response form, indicating their willingness to participate, discussed further details about what participation would involve and provided the opportunity for questions. For those willing to be interviewed, a copy of the CODE^TM^ questionnaire was sent out and completed prior to the interview. A mutually suitable time and place was arranged for the one-to-one interview to occur. Following written informed consent, a structured cognitive ‘think aloud’ interview was conducted (by researchers experienced in cognitive interviewing or a member of the palliative care team), consisting of the following:

- General questions asking about the layout or structure of the CODE^TM^ questionnaire;
- In-depth questions using the ‘think aloud’ method supported by ‘probes’;
- Opportunities for the participant to raise any other issues that had not been discussed and/or additional questions they perceived were needed.

Specific interview questions for each country were formed from the issues that had arisen during the translation process or from the public engagement events. Each interview was audio-taped after gaining the participant’s permission. Field notes were collated after each interview and where possible, the interviews were transcribed verbatim. Alternatively, the interviews were listened to on several occasions by the research team.

### Analysis and collation of feedback

Interviews were analysed using a thematic approach^21^ by one or more members of the research team within each country and categorised into the following options: clarity, recall, sensitivity and response options. These categories are in keeping with the cognitive question–response model of comprehension, retrieval, judgement and response formulation.^25^ Feedback about CODE^TM^, from both the public engagement events and the cognitive interviews, was collated onto a standardised feedback form (SFF) specifically developed for this project. Based on this feedback, each country’s project lead added to the SFF their conclusion about whether or not each individual CODE^TM^ question should be contained within i-CODE. Project leads were advised that questions regarded as irrelevant or insensitive from a cultural point of view may be legitimate for omission.

### Consensus meeting

To reach consensus about the content of the international (‘i-CODE’) questionnaire, a structured telephone meeting, in keeping with the principles of a nominal group technique,^26^ was held with the participating countries’ project leads (*n* = 8). The meeting was facilitated by the overall project lead (D.F.H.), who was not directly involved in the pre-testing, and the WP lead (C.R.M.). Project leads (within each country) were blinded to others’ decisions while they made their own conclusions, which were submitted prior to the consensus meeting.

The following steps were undertaken within the meeting:

1. The meeting objective was outlined.
2. In turn, each project lead provided a summary of their pre-testing findings and main conclusions.
3. Key discussion points were listed (where there were differing opinions) and a subsequent round of questioning was conducted with voting to reach consensus.

Prior to the meeting, a decision was made that if four or more project leads had concluded that an individual question should be removed, this question would be omitted from the i-CODE questionnaire (with this decision relating to the potential cultural sensitivities that could arise).

## Results

### Translation of ‘CODE’ questionnaire

Translation was undertaken for the three new languages. Specific problems encountered mainly related to the following:

There being no translation for specific English words, for example, no Norwegian equivalent for ‘distressed’ or ‘care’ so an appropriate alternative had to be chosen;
The Portuguese and Spanish language having different forms for masculine and feminine nouns, therefore using the term ‘he/she’ made reading less fluent;
Culturally, there was sometimes a need to use appropriate alternatives to the original words to suit the individual language better, for example, ‘banheiro’ or ‘bathroom’ rather than ‘toilet’ in Portuguese.

Issues raised during the translation process were taken forward to be addressed within the subsequent pre-testing stages.

### Public engagement events and cognitive interviews with bereaved relatives

These activities were undertaken between March and July 2017, except Germany and Poland, who conducted their cognitive interviews prior to this period. For each country, a group workshop was facilitated with PPI representatives (Table 1), with 48 individuals participating within the project as a whole. Most participants were female and had a volunteer role. Identified issues were either brought forward for discussion at the consensus meeting or further assessed using cognitive interviews conducted with bereaved relatives (Table 2). Interviews generally lasted between 18 and 60 min. Views about the proposed method of recruitment in the future study were recorded, where appropriate (Table 3). From the 35 cognitive interviews, in addition to the PPI views, individual country feedback was collated and categorised (Table 3). Although the intent was for cognitive interviews to be conducted within Brazil, delays in obtaining ethical approval meant these were not able to be undertaken. Individual project lead reviewed the overall feedback and concluded for each individual question whether it should be contained within i-CODE.

**Table 1.** Demographic details of participants within public engagement events.

Language (Country)	No.	Gender	Role
English (United Kingdom)	9	5 females 4 males	Care of the dying volunteer (*n* = 4) Palliative care institute or ‘People’s Voice’ patient and public representative (*n* = 5)
German (Germany)	9	7 females 2 males	Palliative care unit volunteers (*n* = 4); hospice volunteers (*n* = 2); hospital volunteer (*n* = 1); Turkish volunteers (*n* = 2; nurse = 1, family carer = 1)
Norwegian (Norway)	5	4 females 1 male	Hospital volunteers (*n* = 4); recruited from the general public (*n* = 1)
Polish (Poland)	5	3 females 2 males	Care of the dying volunteers (*n* = 5)
Portuguese (Brazil)	5	3 females 2 males	Hospital patient and public representatives (*n* = 3)^a^; Sumaré State Hospital staff (*n* = 2)
Spanish (Uruguay)	6	5 females 1 male	Care of the dying volunteer (*n* = 4); volunteer opinion group members (*n* = 2)
Spanish (Argentina)	6	5 females 1 male	Care of the dying volunteer (*n* = 4); patient and public representative (*n* = 2)

a These individuals have paid employment within the hospital (within maintenance, domestic cleaning and administrative teams) but are not directly clinically based.

**Table 2.** Demographic details of participants within cognitive interviews.^a^

Language (Country)	No.	Gender	Age range (years)	Relationship to patient
German (Germany)	15	11 females 4 males	20–79	Spouse/partner: 8 Child: 4 Parent: 1 Other: 2 (niece, divorcee)
Norwegian (Norway)	5	3 females 2 males	40–69	Spouse/partner: 3 Child: 2
Polish (Poland)	5	2 females 3 males	30–80	Spouse/partner: 3 Child: 2
Spanish (Uruguay)	5	5 females	55–69	Parent: 1 Child: 3 Niece: 1
Spanish (Argentina)	5	4 females 1 male	40–69	Spouse/partner: 1 Child: 4

a Due to delay in obtaining ethical approval for the study, cognitive interviews were not performed in Brazil.

**Table 3.** Main feedback about CODE^TM^ questionnaire from participants within public engagement events and cognitive interviews.^a^

Language (Country)	Clarity	Recall	Sensitivity	Response options	Other comments (methods; additional items)
German(Germany)	Queried about the meaning of the type of ‘restlessness’.Preamble to the symptom control section modified to emphasise asking about respondents’ perceptions.Could find it difficult to separate ‘spiritual’ and ‘religious’ and often thought of them together.	Recall perceived as easy as participants had vivid memories of this time.	Deciding about whether it was the ‘right’ place to die was quite challenging.Rephrased question to emphasise ‘the right place under the circumstances given’.	Could be difficult to assess pain in others so responding to questions about this is challenging.Simplified response option from ‘No, s/he did not appear to be in pain’ to ‘No’.Discussed option of adding ‘not applicable’ response options for religious and spiritual needs.Changes needed to ethnicity questions to be culturally relevant.	Additional sub-question requested regarding who told the bereaved relative that the patient was likely to die.
Norwegian(Norway)	Easy to understand.	No specific issues raised.	Keep second page of questionnaire blank to avoid impression of question overload.Asking about the *‘right place’* can be sensitive if their wish was not fulfilled.	Generally, good response options.Keep consistency of ordering response options, i.e., positive to negative.Requested for some additional response options if this was possible.Changes needed to ethnicity and religious affiliation questions to be culturally relevant.	Appeared culturally appropriate.Additional 2 questions added to ask about advance care planning.
Polish(Poland)	No specific issues raised.	No specific issues raised.	Asking about ethnicity can be sensitive.	Consider simplifying response options for spiritual needs and ‘Friends and Family’ questions.Changes needed to ethnicity and religious affiliation questions to be culturally relevant.	
Portuguese(Brazil)a	Consider additional text in preamble about the term ‘emotional support’.	No specific issues raised.	No specific issues raised.	Changes needed to ethnicity questions to be culturally relevant.	
Spanish(Argentina)	Participants did not always understand the term ‘restlessness’ although were still able to answer the question appropriately.Most participants perceived that ‘spiritual’ and ‘religious’ needs were synonyms but others perceived these were different kinds of needs and wondered whether separate questions were needed.Some difficulty understanding the term ‘noisy rattle to his/her breathing’.	It could be challenging limiting recall solely to the last days of life.Some difficulty recalling information to answer the questions about ‘noisy rattle to his/her breathing’ and discussions about what to expect when the patient was dying.Participants specifically recalled differences between care from the palliative care team and other healthcare teams.	In Latin American culture, death is a moment rather than a process. So, some questions asking about changes to expect when someone is dying were more difficult to understand.Also ‘dying patient’ may need different description.Some participants felt uncomfortable about assessing whether the patient had died in the ‘right place’ and alternative terminology was adopted.The ‘right place’ was perceived as the place the patient would receive the best care and/or a place convenient to the family (not necessarily the patients’ preferred place of care.Some participants perceived the statement about ‘religious or spiritual’ needs as an assumption they had strong needs in this area.Culturally, not appropriate to ask about ethnicity groups.	Participants expressed preference for ‘yes/no’ format or multiple choice rather than Likert-type responses.Changes needed to ethnicity questions to be culturally relevant.	
Spanish(Uruguay)	The term ‘bed area’ does not have a clear translation and another appropriate term would be needed.Queried whether the question asking where the patient died was needed (as all patients within this study had died in hospital).	Some participants would prefer the time frame to be greater than last days of life.	One participant found the question asking about ‘religious or spiritual’ needs challenging.	Suggestions to use additional response options to help differentiate between different teams, different ward areas and impact of the palliative care team.Questioned whether an additional response option was required for the question asking about support at the time of death, ‘Not applicable, I didn’t have any contact with the healthcare team at the actual time of his/her death’.Changes needed to ethnicity and religious affiliation questions to be culturally relevant.	Some additional areas for potential questions include staff training, use of sedation and evaluation of the palliative care team.
Englisha(United Kingdom)	Some specific terms prompted discussion, e.g., ‘noisy rattle to the breathing’ and ‘religious’ although no specific recommended changes were suggested.	No specific issues raised.	One participant wondered whether ‘preferred place’ rather than ‘right place’ would be more sensitive.	No specific issues raised.Appreciated the free-text section to allow ‘individual stories’ to be heard.	Supportive of method of approach – initial study information to next-of-kin when collect death certificate.

a Only from public engagement event.

Key areas of commonality across all countries included CODE^TM^ being perceived as clear, comprehensive and user-friendly in terms of completion. All countries (except the United Kingdom) reported that changes were needed to the question items relating to ethnicity. The most culturally challenging areas were raised by the Spanish participants, as death is perceived as a ‘moment’ rather than a ‘process’ and this impacted question items relating to communicating what to expect when someone is dying.

### Consensus meeting

The main results were as follows:

1. From the pre-testing results, there were no specific question items that four or more project leads thought should be excluded from the CODE^TM^ questionnaire. One country suggested that we could remove the question E26 asking about the place of death since all the patients should have died in hospital. Subsequent discussion deemed this was an extra level of checking inclusion criteria and allowed CODE^TM^ to be used in all care settings.
2. The demographic details section (ethnicity and religious affiliation options) within CODE^TM^ needed to be revised for each country to ensure it was relevant and sensitive (Table 4).
3. Specific additional questions to help differentiate between the impacts of the SPC team and ward areas were not added as this could be conducted at the subsequent analysis stage (and we wished to minimise participant response burden). As Norway was conducting explicit work relating to advance care planning, additional questions relating to this topic were added, but these were not thought to be ‘core’ questions relevant for all countries. Three countries wished to add a free-text question asking who had informed the participant that their family members/friend was likely to die soon (question E23).
4. Re-ordering, where appropriate, of response options was conducted to keep consistency throughout the questionnaire.
5. Additional response options, although preferred in some countries, were not included, preserving consistency across all languages.
6. Section D (‘Emotional and spiritual support’) raised a number of issues and a decision was made to add additional information into the preamble section to help provide further clarity.
7. A more culturally appropriate translation was needed for some specific English words while still retaining the intended meaning, for example, ‘*right place’ in terms of place of death*.

**Table 4.** Changes to the demographic details section of the CODE^TM^ questionnaire for each country.

Demographic details in original English version of CODE^TM^	Respondent’s relationship to deceased	Respondent’s age group (years)	Respondent’s ethic group	Respondent’s gender	Respondent’s religious affiliation	Patient’s illness	Patient’s age group (years)	Patient’s ethic group	Patient’s gender	Patient’s religious affiliation
Germany	Changed response option from ‘parent’ to ‘mother/father’	Added ‘90–99’ and ‘100+’ categories	Changed to: ‘What is your nationality?’ ‘Do you have a migrant background?’ Used free-text response	Used male and female versions of questionnaire	Reduced response options to: ‘Protestant’ ‘Roman-Catholic’ ‘Muslim’ ‘None’ ‘Other’ (please specify)	Added response option: ‘Stroke’	Added ‘90–99’ and ‘100+’ categories	Changed to: ‘What was his/her nationality?’ ‘Did he/she have a migrant background?’ Used free-text response	Used male and female versions of questionnaire	Reduced response options to: ‘Protestant’ ‘Roman-Catholic’ ‘Muslim’ ‘None’ ‘Other’ (please specify)
Norway	No change	Added ‘90+’ category	Changed to: ‘What is your nationality?’ ‘Do you have an immigrant background?’ Used free-text response	No change	Changed to: ‘What is your faith/principal affiliation?’ Added response option: ‘Humanist’	Added response option: ‘Stroke/cerebral haemorrhage’	Added ‘90+’ category	Changed to: ‘What was his/her nationality?’ ‘Did s/he have an immigrant background?’ Used free-text response	No change	Changed to: ‘What was his/her faith/principal affiliation?’ Added response option: ‘Humanist’ ‘Don’t know’
Poland	No change	Added ‘90+’ category	Changed to: ‘Please indicate which ethnic group you belong to: ‘White’ ‘Other (please state)’	No change	Response options changed to: ‘None’ ‘Christian (Catholic)’ ‘Christian (other denominations)’ ‘Other (please state)’	Added response options: ‘Cerebral ischaemia/Stroke’ ‘Other (please state)	Added ‘90+’ category	Changed to: ‘Please indicate which ethnic group your closest relative belonged to: ‘White’ ‘Other (please state)’	No change	Response options changed to: ‘None’ ‘Christian (Catholic)’ ‘Christian (other denominations)’ ‘Other (please state)’
Brazil	No change	Added ‘90+’ category	Changed response options to: ‘White’ ‘Black’ ‘Asian’ ‘Mixed other’ ‘Indian’ ‘None of these’	No change	Changed response options to: ‘Catholic’ ‘Evangelical’ ‘Spiritist’ ‘Jehovah’s Witness’ ‘Buddhist’ ‘Candomble’ ‘Jewish’ ‘Any other religion’	No change	Added ‘90+’ category	Changed response options to: ‘White’ ‘Black’ ‘Asian’ ‘Mixed other’ ‘Indian’ ‘None of these’	No change	Changed response options to: ‘Catholic’ ‘Evangelical’ ‘Spiritist’ ‘Jehovah’s Witness’ ‘Buddhist’ ‘Candomble’ ‘Jewish’ ‘Any other religion’
Argentina and Uruguay	No change	Added ‘90+’ category	Changed to: ‘What is your nationality/cultural background ethnic group?’ ‘Do you have an immigrant background?’ ‘If yes, from which country/countries?’ Used free-text response	No change	Changed to: ‘What is your faith/principal religion?’ Removed response option: ‘Sikh’	Added response option: ‘Stroke/cerebral haemorrhage’	Added ‘90+’ category	Changed to: ‘What was his/her nationality/cultural background ethnic group?’ ‘Did s/he have an immigrant background?’ ‘If yes, from which country/countries?’ Used free-text response	No change	Changed to: ‘What was his/her faith/principal religion?’ Removed response option: ‘Sikh’

## Discussion

### Main findings

Overall, we have developed a common, core international questionnaire (‘i-CODE’) with key questions pertaining to the quality of care for those who are dying. In addition, we have culturally adapted versions, combining the views of PPI representatives, and, with the exception of Brazil, bereaved relatives’ views for each language. On an international basis, the i-CODE questionnaire appears to have good face and content validity. As individual questions appeared to be culturally relevant across all seven participating countries, the next part of the research process – a cross-sectional survey with bereaved relatives – is feasible and a transnational comparison of results is possible. Further assessment of the psychometric properties of the CODE^TM^ questionnaire will be facilitated during the next steps of this research.

The feedback from the PPI events and cognitive interviews was beneficial in terms of refining specific wording of questions to help with clarity and sensitivity. In particular, suggestions regarding the wording of the ‘demographic details’ section of the CODE^TM^ questionnaire were especially pertinent to ensure that ethnicity and religious affiliations were culturally appropriate.

### Strengths and limitations

In constructing the international development of the CODE^TM^ questionnaire, we have been mindful of the value and benefit from both PPI representatives and having direct feedback from our future target audience, the bereaved relatives. Hence, active engagement with both parties was key, and the bringing together or ‘triangulation’ of different information sources within each participating country enhanced the development process. Public involvement in research is recognised to improve the ‘relevance and overall quality of the research, by ensuring it focuses on issues of importance to patients’.^27^ One key example was the English PPI input into the methodology, that is, providing initial information about the study to the next-of-kin when they collect the death certificate, which was subsequently discussed at the ethical review committee. The value of cognitive interviewing within palliative care research is established^28,29^ and recommended as a standard part of piloting instruments.^28^ We were able to undertake cognitive interviews in all bar one country, helping highlight issues and concerns standard pilot testing may not identify. Our main limitations were as follows:

1. Our participating numbers for each country were relatively small, although they do meet current recommendations for cognitive interviews (5–15 respondents).^30^ In addition, efforts were made to warrant access to specific groups to provide a broad perspective, for example, migrants in Germany. The predominance of female participants is notable, although there was diversity in terms of age groups and roles/relationships to the deceased patient.
2. Due to ethical restrictions, Brazil was unable to conduct cognitive interviews and their public engagement events also included two healthcare professionals. This may limit the extent to how robust the Portuguese version of CODE^TM^ is in terms of face and content validity. Further reassessment and refinement may subsequently be required and undertaking cognitive interviews at a future date would be recommended.
3. Although the cognitive interviews were conducted by external researchers where possible, some were undertaken by members of the SPC team which may have influenced responses or judgement. The project leads for each country sometimes had dual roles that could have introduced a degree of bias in how results were interpreted. Criteria were set prior to the consensus meeting, however, regarding what would constitute exclusion of a specific question. Finally, WP1 lead was responsible for the original development of ‘CODE’^TM^, potentially influencing perspectives. This person’s expertise in pre-testing survey methods, however, and the potential ethical issues that could arise, was thought to be beneficial to the overall project conduct.
4. Being able to transcribe all the interviews verbatim would have enhanced the detail and depth of the analysis.
5. Due to the funding remit, CODE^TM^ was only tested with those who had a family member dying from cancer. However, CODE^TM^ can be used to assess quality of care for those who died from illnesses other than cancer, so this may limit the generalisability of this pre-testing work.

### What this research adds

To our knowledge, this is the first time within palliative care that pre-testing a post-bereavement questionnaire across seven different countries has been undertaken. In one study, it was used to bring together the knowledge from two European countries simultaneously, for a palliative patient–related outcome measure.^31^ Within other fields of research, using cognitive interviewing consecutively with a number of different languages is more established.^32^ Challenges with cross-national cognitive interviewing are recognised.^30^ For this study, a balance had to be reached between what was methodologically ideal, and what was practical and feasible within the different countries. For example, our sample selection was purposive, and although a structured approach to the interviews was adopted, we did not use the same standardised probes within all countries. This, however, was to allow for flexibility and ensure that feedback was tailored to the issues most pertinent for that individual language.

Combining both European and Latin American countries, where there is variability as to the extent to which palliative care is established and supported, also provides uniqueness. There are potentially different views on what a ‘good death’ constitutes depending on the cultural environment. Many studies focus on the Western society view of what remains important as people approach the end of life.^33,34^ The fact that no individual question was removed from CODE^TM^ supports the questionnaire’s content as representative of key concepts of care for the dying that are internationally relevant and applicable. In addition, the importance of ensuring the family is part of the ‘unit of care’ when evaluating the quality of dying and death is recognised.^35^ This would be in keeping with the fundamental conceptual design for CODE^TM^ where both patient care and family-carer support are assessed.

In keeping with the growing evidence-base, in all seven countries, research about the dying phase of life is an internationally accepted important issue. And, when approached in a sensitive, appropriate manner, there is great willingness for lay people including bereaved relatives to contribute to research. The i-CODE questionnaire is currently being used within the seven countries to conduct a post-bereavement survey with plans for further psychometric testing and refinement to be undertaken within this next stage. This will provide a potential model for a cross-sectional survey to inform how best to meet the care for those in the last days of life.

## Supplemental Material

818299_supp_mat_ – Supplemental material for Assessing quality of care for the dying from the bereaved relatives’ perspective: Using pre-testing survey methods across seven countries to develop an international outcome measure Click here for additional data file. Supplemental material, 818299_supp_mat_ for Assessing quality of care for the dying from the bereaved relatives’ perspective: Using pre-testing survey methods across seven countries to develop an international outcome measure by Catriona Rachel Mayland, Christina Gerlach, Katrin Sigurdardottir, Marit Irene Tuen Hansen, Wojciech Leppert, Andrzej Stachowiak, Maria Krajewska, Eduardo Garcia-Yanneo, Vilma Adriana Tripodoro, Gabriel Goldraij, Martin Weber, Lair Zambon, Juliana Nalin Passarini, Ivete Bredda Saad, John Ellershaw and Dagny Faksvåg Haugen in Palliative Medicine
