# Supplementary material for: Assessing quality of care for the dying from the bereaved relatives’ perspective: Using pre-testing survey methods across seven countries to develop an international outcome measure
Source: Palliat Med. 2019 Jan 10;33(3):357–68. doi: 10.1177/0269216318818299 (PMC6376606; doi:10.1177/0269216318818299)
Supplement: 818299_supp_mat_ – Supplemental material for Assessing quality of care for the dying from the bereaved relatives’ perspective: Using pre-testing survey methods across seven countries to develop an international outcome measure [file 818299_supp_mat_.pdf]

CODE™

Care Of the Dying Evaluation

Palliative Care Institute Liverpool

You have been sent this questionnaire as you are registered as the next of kin to a patient who died in our organisation. This questionnaire is asking about care provided in the last hours and days of their life and your experience of that time. Although we know the patient's name, to ensure confidentiality we have used the phrase 'he/she' in the questionnaire.

We realise this questionnaire may bring back strong memories and emotions and that reading it for the first time may be difficult. You may wish to wait and find someplace quiet to read the questionnaire. If you feel upset or distressed in any way, you do not have to continue with the questionnaire and can stop at any time. By completing the questionnaire, you show that you consent to participate in the project.

Instructions for completion

When answering the questions, we would like you to focus on the last **two days** of his/her life. Please fill in as much of the questionnaire as you can.

As you go through the questionnaire, please follow the instructions and answer the questions by crossing the most appropriate box, like this: ☒

Here is an example question:

Please look at the following statements and cross the ☒ answer box that corresponds most with your opinion.

1. There was enough help available to meet his/her personal care needs, such as washing, personal hygiene and toileting needs.

Strongly agree	☐
Agree	X
Neither agree nor disagree	☐
Disagree	☐
Strongly disagree	☐

If, in your opinion, you agreed that there was enough help to meet his/her personal care needs, you would cross the 'Agree' box as in the example. If you would rather not or cannot answer one of the questions, please go onto the next one. Your answers will be treated as strictly confidential. Individuals will not be identifiable in the reports we write.

Unique Identifier Number:

Copyright Palliative Care Institute Liverpool, University of Liverpool

Section A: The care received from the nurses & doctors

These questions are concerned with the general care he/she received from the doctors and nurses and, where appropriate the environment in which this care was delivered. The questions apply to the last **two days** of his/her life and relates to the doctors and nurses (including healthcare assistants and / or care agency staff) who were most involved with his/her care during this time.

Please look at the following statements and cross ☒ the answer box that corresponds most with your opinion.

1. There was enough help available to meet his/her personal care needs, such as washing, personal hygiene and toileting needs.

- Strongly agree ☐
- Agree ☐
- Neither agree nor disagree ☐
- Disagree ☐
- Strongly disagree ☐

2. There was enough help with nursing care, such as giving medicines and helping him/her find a comfortable position in bed.

- Strongly agree ☐
- Agree ☐
- Neither agree nor disagree ☐
- Disagree ☐
- Strongly disagree ☐

3. The bed area and surrounding environment was comfortable for him/her.

- Not applicable, he/she died at home ☐
- Strongly agree ☐
- Agree ☐
- Neither agree nor disagree ☐
- Disagree ☐
- Strongly disagree ☐

4. The bed area and surrounding environment had adequate privacy for him/her.

- Not applicable, he/she died at home ☐
- Strongly agree ☐
- Agree ☐
- Neither agree nor disagree ☐
- Disagree ☐
- Strongly disagree ☐

5. In your opinion, how clean was the ward area that he/she was in?

- Not applicable, he/she died at home ☐
- Very clean ☐
- Fairly clean ☐
- Not at all clean ☐

6. Did you have confidence and trust in the nurses who were caring for him/her?

- Yes, in all of them ☐
- Yes, in some of them ☐
- No, not in any of the nurses ☐

7. Did you have confidence and trust in the doctors who were caring for him/her?

- Yes, in all of them ☐
- Yes, in some of them ☐
- No, not in any of the doctors ☐

Unique Identifier Number:

Copyright Palliative Care Institute Liverpool, University of Liverpool

8. The nurses had time to listen and discuss his/her condition with me.

- Strongly agree ☐
- Agree ☐
- Neither agree nor disagree ☐
- Disagree ☐
- Strongly disagree ☐

9. The doctors had time to listen and discuss his/her condition with me.

- Strongly agree ☐
- Agree ☐
- Neither agree nor disagree ☐
- Disagree ☐
- Strongly disagree ☐

Section B: The control of pain & other symptoms

These questions are concerned with the symptoms he/she had and the care he/she received during the last **two days** of his/her life.

10. In your opinion, during the last two days, did he/she appear to be in pain?

- Yes, all of the time ☐
- Yes, some of the time ☐
- No, he/she did not appear to be in pain ☐

11. In your view, did the doctors and nurses do enough to help relieve the pain?

- Yes, all of the time ☐
- Yes, some of the time ☐
- No, not at all ☐
- Not applicable, he/she was not in pain ☐

12. In your opinion, during the last two days, did he/she appear to be restless?

- Yes, all of the time ☐
- Yes, some of the time ☐
- No, he/she did not appear to be restless ☐

13. In your view, did the doctors and nurses do enough to help relieve the restlessness?

- Yes, all of the time ☐
- Yes, some of the time ☐
- No, not at all ☐
- Not applicable, he/she was not restless ☐

14. In your opinion, during the last two days, did he/she appear to have a 'noisy rattle' to his/her breathing?

- Yes, all of the time ☐
- Yes, some of the time ☐
- No, he/she did not have a 'noisy rattle' to the breathing ☐

15. In your view, did the doctors and nurses do enough to help relieve the 'noisy rattle' to his/her breathing?

- Yes, all of the time ☐
- Yes, some of the time ☐
- No, not at all ☐
- Not applicable, there was no 'noisy rattle' to his/her breathing ☐

Unique Identifier Number:

Copyright Palliative Care Institute Liverpool, University of Liverpool

Section C: Communication with the healthcare team

The following questions are about the communication that you, your family members and friends received from the healthcare team who were most involved with his/her care in the last **two days** of his/her life. By **'healthcare team'**, we mean the doctors, the nurses and any other member of staff who may have been involved in his/her care such as a social worker or a chaplain.

16. During the last two days, how involved were you with the decisions about his/her care and treatment?

- Very involved ☐
- Fairly involved ☐
- Not involved ☐

17. Did any of the healthcare team discuss with you whether giving fluids through a 'drip' would be appropriate in the last two days of life?

- Yes ☐
- No ☐
- Don't know ☐

18. Would a discussion about the appropriateness of giving fluids through a 'drip' in the last two days of life have been helpful?

- Yes ☐
- No ☐
- Not applicable, we had these types of discussions ☐

19. Did the healthcare team explain his/her condition and/or treatment in a way you found easy or difficult to understand?

- Very easy ☐
- Fairly easy ☐
- Fairly difficult ☐
- Very difficult ☐
- They did not explain his/her condition or treatment to me ☐

Unique Identifier Number:

Copyright Palliative Care Institute Liverpool, University of Liverpool

Section D: The emotional & spiritual support provided by the healthcare team

The following questions are about the emotional and spiritual support that was provided to you and your family member or friend by the healthcare team in the last **two days** of his/her life. By **'spiritual support'**, we mean support relating to important personal beliefs. These beliefs may be connected with a specific religion but may also be personal beliefs about what life means, what provided you or your family member / friend with hope and helped you cope.

20. How would you assess the overall level of emotional support given to you by the healthcare team?

- Excellent ☐
- Good ☐
- Fair ☐
- Poor ☐

21. Overall, his/her religious or spiritual needs were met by the healthcare team.

- Strongly agree ☐
- Agree ☐
- Neither agree nor disagree ☐
- Disagree ☐
- Strongly disagree ☐

22. Overall, my religious or spiritual needs were met by the healthcare team.

- Strongly agree ☐
- Agree ☐
- Neither agree nor disagree ☐
- Disagree ☐
- Strongly disagree ☐

Section E: The circumstances surrounding his/her death

The following questions are about the circumstances surrounding his/her death, and your feelings about the way in which the healthcare team treated you and your family member at this time. By **'healthcare team'**, we mean the doctors, the nurses and any other member of staff who may have been involved in his/her care such as a social worker or a chaplain.

23. Before he/she died, were you told he/she was likely to die soon?

- Yes ☐
- No ☐

24. Did a member of the healthcare team talk to you about what to expect when he/she was dying (e.g. symptoms that may arise)?

- Yes ☐
- No ☐

Unique Identifier Number:

Copyright Palliative Care Institute Liverpool, University of Liverpool

25. Would a discussion about what to expect when he/she was dying have been helpful?

Yes ☐
No ☐
Not applicable, we had these types of discussions ☐

26. Where did he/she die?

At home ☐
In a hospital ☐
In a hospice ☐
In a care home / nursing home ☐
Other (please specify) ☐

____

27. In your opinion did he/she die in the right place?

Yes, it was the right place ☐
No, it was not the right place ☐
Not sure ☐
Don't know ☐

28. I was given enough help and support by the healthcare team at the actual time of his/her death.

Strongly agree ☐
Agree ☐
Neither agree nor disagree ☐
Disagree ☐
Strongly disagree ☐

29. After he/she had died, did individuals from the healthcare team deal with you in a sensitive manner?

Yes ☐
No ☐
Not applicable, I didn't have any contact with the healthcare team ☐

Section F: Overall impressions

The following questions are about your overall impression of the care he/she received in the last **two days** of life and your experiences during that time.

30. How much of the time was he/she treated with respect and dignity in the last two days of life?

Please answer for both doctors and nurses

	Doctors	Nurses
Always	☐	☐
Most of the time	☐	☐
Some of the time	☐	☐
Never	☐	☐
Don't know	☐	☐

Unique Identifier Number:

Copyright Palliative Care Institute Liverpool, University of Liverpool

31. Overall, in your opinion, were you adequately supported during his/her last two days of life?

Yes ☐
No ☐

32. How likely are you to recommend our Organisation to friends and family?

Extremely likely ☐
Likely ☐
Neither likely nor unlikely ☐
Unlikely ☐
Extremely unlikely ☐
Don’t know ☐

Although the focus of this questionnaire has very much been on his/her last days of life, we appreciate there may be other aspects of care or support prior to this time, which you wish to feedback. Please feel free to comment, if you wish to, on any aspect of the overall care and support received:

Unique Identifier Number:

Copyright Palliative Care Institute Liverpool, University of Liverpool

Section G: Information about you and your relative or friend

We would like to know a little more about you and your relative or friend. This will help us make further use of the information you give us and will remain strictly confidential

33. What was your relationship to him/her? Were you his/her:

- Husband / Wife / Partner ☐
- Son / Daughter ☐
- Brother / Sister ☐
- Son-in-law / Daughter-in-law ☐
- Parent ☐
- Friend ☐
- Neighbour ☐
- Staff in nursing or residential home ☐
- Warden (sheltered accommodation) ☐
- Other ☐

34. What is your age?

- 18 – 19 ☐
- 20 – 29 ☐
- 30 – 39 ☐
- 40 – 49 ☐
- 50 – 59 ☐
- 60 – 69 ☐
- 70 – 79 ☐
- 80 – 89 ☐
- 90+ ☐

35. Please could you indicate which ethnic group you belong to:

- White British ☐
- Mixed white / black Caribbean ☐
- White Irish ☐
- Mixed white / black African ☐
- White other ☐
- Mixed white / Asian ☐
- Indian ☐
- Mixed other ☐
- Pakistani ☐
- Black Caribbean ☐
- Bangladeshi ☐
- Black African ☐
- Chinese ☐
- Asian other ☐
- Black other ☐
- Other ☐

36. Are you:

- Male ☐
- Female ☐

37. What is your religious affiliation?

- None ☐
- Christian (all denominations) ☐
- Hindu ☐
- Buddhist ☐
- Jewish ☐
- Muslim ☐
- Sikh ☐
- Any other religion ☐

38. This question is about the illnesses he/she may have had in the last days and hours of life. Here is a list of illnesses which often affect people towards the end of life. Please cross all the illnesses he/she had in the last days of life.

- Cancer (including leukaemia and lymphoma) ☐
- Heart failure ☐
- COPD (chronic obstructive airways disease) ☐
- End-stage renal (or kidney) disease ☐
- Dementia ☐
- Motor Neurone Disease ☐
- Don’t know ☐
- Something else ☐

Unique Identifier Number:

Copyright Palliative Care Institute Liverpool, University of Liverpool

39. What was his / her age when she/he died?

- 18 – 19 ☐
- 20 – 29 ☐
- 30 – 39 ☐
- 40 – 49 ☐
- 50 – 59 ☐
- 60 – 69 ☐
- 70 – 79 ☐
- 80 – 89 ☐
- 90+ ☐

40. Please could you indicate which ethnic group he/she belonged to:

- White British ☐
- Mixed white / black Caribbean ☐
- White Irish ☐
- Mixed white / black African ☐
- White other ☐
- Mixed white / Asian ☐
- Indian ☐
- Mixed other ☐
- Pakistani ☐
- Black Caribbean ☐
- Bangladeshi ☐
- Black African ☐
- Chinese ☐
- Asian other ☐
- Black other ☐
- Other ☐

41. Was he/she:

- Male ☐
- Female ☐

42. What was his/her religious affiliation?

- None ☐
- Christian (all denominations) ☐
- Hindu ☐
- Buddhist ☐
- Jewish ☐
- Muslim ☐
- Sikh ☐
- Any other religion ☐

Thank you very much for taking the time to complete this questionnaire

We would be very grateful if you could return it to us using the pre-paid envelope enclosed.

Unique Identifier Number:

Copyright Palliative Care Institute Liverpool, University of Liverpool
